# Toll-Like Receptor 4 Expression on Lymphoma Cells Is Critical for Therapeutic Activity of Intratumoral Therapy With Synthetic TLR4 Agonist Glucopyranosyl Lipid A

**DOI:** 10.3389/fonc.2020.01438

**Published:** 2020-08-19

**Authors:** Hailing Lu, Alec Betancur, Michael Chen, Jan H. ter Meulen

**Affiliations:** Immune Design Corp., Seattle, WA, United States

**Keywords:** TLR4, GLA, B-cell lymphoma, intratumoral treatment, tumor microenvironment

## Abstract

Intratumoral (IT) injections of Glucopyranosyl lipid A (G100), a synthetic toll-like receptor 4 (TLR4) agonist formulated in a stable emulsion, resulted in T-cell inflammation of the tumor microenvironment (TME) and complete cure of 60% of mice with large established A20 lymphomas. Strong abscopal effects on un-injected lesions were observed in a bilateral tumor model and surviving mice resisted a secondary tumor challenge. Depletion of CD8 T-cells, but not CD4 or NK cells, abrogated the anti-tumor effect. Unexpectedly, TLR4 knock-out rendered A20 tumors completely non-responsive to G100. *In vitro* studies showed that GLA has direct effect on A20 cells, but not on A20 cells deficient for TLR4. As shown by genotyping and phenotyping analysis, G100 strongly activated antigen presentation functions in A20 cells *in vitro* and *in vivo* and induced their apoptosis in a dose dependent manner. Similarly, the TLR4 positive human mantle cell lymphoma line Mino showed *in vitro* activation with G100 that was blocked with an anti-TLR4 antibody. In the A20 model, direct activation of B-lymphoma cells with G100 is sufficient to induce protective CD8 T-cell responses and TLR4 expressing human B-cell lymphomas may be amenable to this therapy as well.

## Introduction

Only a minority of cancer patients currently benefits from immunotherapeutic interventions aimed at rescuing functional T-cells responses through modulation of immune check points, and this resistance is at least partly be due to the immunosuppressive nature of the tumor microenvironment (TME) (1). Immune recognition of tumor cells and priming of CD8 T-cell responses in the tumor draining lymph node requires processing and presentation of tumor antigens by cross-presenting dendritic cells, coupled with their activation by danger signals, and secretion of type 1 interferons (2, 3). Therefore, therapies that are capable of inducing proinflammatory changes in the TME, activating antigen presenting cells and inducing immune responses should have significant therapeutic potential on their own and in combination with other immunotherapies (4, 5). To this end, several agents designed to modify the TME are currently under clinical investigation, including cytokines, oncolytic viruses, toll-like receptor (TLR) 3, 7/8 and 9 agonists, RIG-I agonists and others (6, 7). While some of these treatment modalities have shown impressive levels of efficacy in murine models, notably agonists of the stimulator of interferon genes (STING) pathway, activity in the clinic has been rather modest to date, especially in solid tumors with low levels of pre-existing T-cell inflammation.

Toll-like receptors (TLRs) belong to the families of pattern recognition receptors (PRRs), and recognize conserved microbial components, which are referred to as pathogen-associated molecular patterns (PAMPs), as well as endogenous danger signals, known as damage-associated molecular patterns (DAMPs). Common TLR-activating PAMPs include viral and bacterial nucleic acids (signaling trough TLR3, TLR7/8, or TLR9), flagellin (a TLR5 agonist), as well as lipopolysaccharide (LPS), lipoteichoic acid, and mannans (which signal through TLR2 or TLR4). Endogenous TLR-activating DAMPs are nucleic acids and the nuclear non-histone protein high mobility group box 1 (HGMB1). TLR control the activation, maturation and immunological functions of immune cells and are as a family either located in the plasma membrane (e.g., TLR2, TLR4, TLR5) or in endosomal membranes (e.g., TLR3, TLR7/8, and TLR9), of macrophages, dendritic cells (DCs), B cells, and natural killer (NK) cells, as well as some non-immune cells including epithelial cells, fibroblasts and malignant cells. Clinical trials are currently focused on evaluating systemic treatment with TLR3 agonists (Hiltonol™, Ampligen™, BO-112™) in combination with standard of care, or with immune checkpoint inhibitors, vaccines, cytokines or other treatments in a variety of solid tumors (colorectal carcinoma, breast, lung, prostate cancer and others), as well as hematological malignancies (MM, AML). TLR7/8 agonists (Imiquimod, Motolimod) are being used topically in a number of solid tumors (squamous cell carcinoma, melanoma, anal carcinoma, cutaneous T-cell lymphoma, and others), and a TLR9 agonist (SD-101) is being evaluated for intratumoral treatment of hematological malignancies (follicular lymphoma) and some solid tumors (prostate and others) (8). Toll-like receptor 4 (TLR4) is part of a cell surface receptor complex that recognizes lipopolysaccharide (LPS) and is expressed on professional antigen-presenting cells (APCs), such as dendritic cells (DC), monocytes, macrophages, and activated B cells, as well as some non-immune cells, including epithelium, endothelium, and smooth muscle (9). Activation of TLR4 on APC results in stimulation of antigen presentation, upregulation of costimulatory molecules such as CD40 and CD80, and secretion of inflammatory cytokines, including IL6 and IL12, and type I interferons (IFN) (10). In addition, TLR4 agonists have recently been shown to signal through an intracellular pathway which leads to activation of the non-canonical inflammasome with secretion of interleukin 1 beta (IL-1β) and IL-18 (11). Glucopyranosyl lipid A (GLA) is a synthetic TLR4 agonist that potently activates dendritic cells and has shown a very good safety and efficacy profile as an infectious disease vaccine adjuvant in several phase 1 and 2 studies (12, 13). Two formulations of GLA have been tested in preclinical and clinical studies, GLA-stable emulsion (SE) and GLA-aqueous formulation (AF). Both formulations have been shown to be potent vaccine adjuvants stimulating both antibody and T cell responses (14, 15). For intratumoral injection, GLA-SE is preferred as the emulsion retains GLA locally at the injection site. Intratumorally injected GLA-SE inflamed the TME in murine melanoma and glioma models, “pulling” antigen-specific, vaccine primed or passively transferred CD8 T-cells into tumors, which resulted in their regression (16). A recent clinical trial showed that intratumoral injection of GLA-SE (termed G100) inflamed the TME of Merkel cell carcinoma patients, with antitumor immune responses and objective tumor responses observed (17).

Using the murine A20 B-cell lymphoma, we investigated the effect of IT G100 on the TME and its dependency on TLR4 expression of the tumor cells.

## Materials and Methods

### Mice

Female Balb/c mice (7~8 weeks old) were purchased from Jackson Laboratory (Bar Harbor, ME) and were maintained under specific pathogen free conditions. All the procedures described in this study were performed in compliance with the local Institutional Animal Care and Use Committee guidelines.

### Reagents and Cell Lines

Fluorochrome-conjugated antibodies targeting mouse CD3, CD4, CD8, B220, NKp46, Foxp3, IFNγ, TNFα, IL2, CD40, CD80, CD86, and TLR4 were obtained from eBiosciences (San Diego, CA). The antibodies used for *in vivo* depletion, anti-CD4 (clone GK1.5) and anti-CD8 (clone 56.3) were purchased from BioXcell (West Lebanon, NH). The A20 cell line, originally derived from B lymphocytes of a naturally occurring reticulum cell sarcoma from an old Balb/c mouse, was obtained from the American Type Culture Collection (ATCC® TIB-208). The A20 cells were expanded in complete RPMI medium (RPMI with 10%FBS, pen/strep, and glutamine) before each tumor inoculation. A biallelic TLR4 knockout A20 cell line was generated using the CRISPR/Cas9 system at GenScript (Piscataway, NJ) and the biallelic gene knockout was confirmed by sequencing analysis. Glucopyranosyl lipid A (GLA, G100) was manufactured and formulated by Immune Design using proprietary methods. For preclinical work, two formulations of GLA were used. For *in vivo* studies, a stable oil-in-water formulation (G100) containing 2 mg/mL GLA in 2% squalene (SE) was adjusted to various GLA concentrations (1, 5, 10 or 20 μg GLA) in 2% SE. GLA-AF (aqueous formulation), which contained the surfactant dipalmitoyl phosphatidylcholine instead of squalene, was adjusted to 5 μg GLA/ml. For *in vitro* experiments, cells were exposed to GLA-AF for 48 h before being analyzed by Flow cytometry for expression of surface markers or analysis for RNA expression profiling. The Mino cell line is a human blood/Mantle cell lymphoma (B cell non-Hodgkin's lymphoma) that was obtained from ATCC (ATCC® CRL-3000).

### A20 Tumor Model

Five million A20 murine lymphoma cells were implanted subcutaneously (s.c.) into Balb/c mice on the right flank (for unilateral tumor model) or on both sides (for a bilateral tumor model, only one tumor injected). Tumor take was close to 100% using this inoculation method. Tumor growth was monitored using a digital caliper every 2–3 days and the tumor size was expressed as surface area (length x width). Mice were sacrificed when the tumor size reached over 200 mm^2^. Intratumoral (IT) injection of G100 or control PBS or SE started on Day 7~9 when the average tumor size was 30~50 mm^2^. The treatment was administered three times per week for a total of 7–9 doses. To investigate the direct effect of GLA without the emulsion on A20 cells with respect to tumor rejection, A20 cells were treated *in vitro* with GLA-AF (5 μg/mL) for 48 h before the cells were harvested and inoculated s.c. into Balb/c mice.

### CD4 and CD8 T Cell Depletion

For selective depletion of CD4 or CD8 T cells, mice received intraperitoneal injection of the depletion antibody (100 μg) for two times at the week before IT G100 treatment and then once per week during treatment. FACS analysis confirmed that the depletion efficiency was more than 95% for both CD4 and CD8 T cells (data not shown).

### Flow Cytometry

Staining of splenocytes was performed as described previously (18). TILs were isolated by centrifugation over Histopaque-1083 (Sigma-Aldrich, St. Louis, MO). For staining of T regulatory cells, splenocytes or TIL were first stained with anti-CD3-eF450/anti-CD4-FITC/anti-CD8-PerCP, and then stained with anti-FoxP3-PE after fixation and permeabilization. For the staining of activation markers and TLR4 expression on A20 cells, the cells were cultured in RPMI medium with or without GLA-AF (5 μg/mL) for 48 h before the cells were harvested for flow cytometry analysis. The cells were then stained with antibodies against CD80, CD86, and CD40, and TLR4 using surface staining. For evaluation of apoptosis and necrosis, cells were stained with an Annexin V (AV) and propidium iodide (PI) staining kit with binding buffer (Invitrogen, Carlsbad, CA). Data acquisition was done on a FACS LSRII flow cytometer (BD Biosciences, San Jose, CA). List mode data were analyzed using the FlowJo software (Tree Star, Ashland, OR).

### Cell Growth Inhibition

The effects of GLA or other TLR agonists on growth of murine or human lymphoma cell lines were evaluated using different methods. In initial studies, cell viability and cell number were enumerated using a Nucleocounter, then a FACS-based high throughput method using LIVE-DEAD-NIR dye (ThermoFisher, Waltham, MA) was employed. For the evaluation of apoptosis and necrosis, cells were stained with AV and PI as described above. To compare the direct effect of different TLR agonists in A20 cells, we cultured A20 cells in 96-well plates (10,000 cells/well) and treated the cells in duplicate with serial dilutions of GLA, TLR3 agonist poly I:C, and TLR9 agonist CpG ODN2006 (InvivoGen, San Diego). After 96 h of incubation, the cells were stained with LIVE-DEAD-NIR dye and analyzed on a flow cytometer. The percentage of viable cells as well as total number of viable cells in each well were analyzed in FlowJo.

### Intracellular Staining (ICS)

ICS was performed using similar method as previously described with modifications (18). The spleens from G100 treated Balb/c mice that rejected A20 tumors and control naïve mice were harvested and homogenized using the AutoMACS tissue dissociator (Miltynei Biotec, San Diego, CA). Red blood cells were lysed by using the ACK lysis buffer from eBiosciences (San Diego, CA). For measurement of cytokine production, splenocytes were stimulated with γ-irradiated A20 tumor cells at a 10:1 ratio in 96-well plates with ~ 500,000 cells per well in complete RPMI medium. Medium containing PMA and ionomycin (eBiosciences) was used as positive control. After 1 h of stimulation, brefeldin A was added to the culture wells and the plate was incubated overnight at 37°C in a humidified CO_2_ incubator. The next day, the cells were stained with anti-CD4-AF-700, anti-CD3-PerCP, anti-CD8-PB, and anti-B220-V500. After surface staining, the cells were fixed with Cytofix buffer (BD Biosciences, San Jose, CA) and then permeabilized with Perm/Wash buffer containing 5% rat serum. Cells were then stained with anti-TNFα-Alexa 488, anti-IFNγ-PE, and anti-IL2-APC for intracellular cytokines. Cells were washed with FACS buffer and analyzed on a 3-laser LSRII cytometer. List mode data were analyzed using the FlowJo 9.0 software.

### Immunohistochemistry (IHC) Analysis

A20 tumors were treated with intratumoral G100 (10 μg) or control PBS every other day. After 3 treatments, tumors were harvested and fixed in 10% formalin. Paraffin embedding, sectioning, and IHC analysis were performed at the Experimental Histology center at Fred Hutchinson Cancer Research Cancer (Seattle, WA). Anti-cleaved caspase 3 antibody (clone D3E9) and anti-Ki67 antibody (clone D3B5) were from Cell Signaling Technology (Danvers, MA).

### RNA Expression Analysis of Murine and Clinical Specimens

A20 tumors were treated with G100 or control PBS every other day for a total of 3 times. Tumors were collected at 2 h after the last treatment and immediately stored in RNAlater (Ambion, Austin, TX). RNA was extracted using the DNA/RNA Allprep kit from Qiagen (Valencia, CA) for analysis with the nCounter® mouse PanCancer Immune Profiling Panel of Nanostring (Seattle, WA), a multiplex gene expression analysis with 770 genes from 24 different immune cell types, common checkpoint inhibitors, CT antigens, and genes covering both the adaptive and innate immune response. The hybridization reaction and scanning of slides was performed by the Genomic Center at Fred Hutchinson Cancer Center, Seattle, WA. The data were normalized and analyzed using nSolver software (Nanostring, Seattle, WA). To study the direct effect of GLA on A20 tumor cells, the cells were cultured in RPMI medium with or without GLA (5 μg/mL) for 48 h before the cells were harvested for RNA extraction and subsequent gene expression analysis using the same Nanostring panel, nCounter® mouse PanCancer Immune Profiling Panel. The expression level of different TLRs in A20 cells was compared by using Nanostring gene expression data normalized with house-keeping genes. Data analysis was done in nSolver using the Advanced Analysis tool with the following parameter setting: remove genes below specified threshold (TRUE); threshold count value (20); covariate (TimePoint); variable type (categorical); reference level (pre-Tx); perform normalization (TRUE); auto-select number of housekeepers (TRUE); perform differential expression testing (TRUE); predictors (TimePoint). TimePoint refers to whether a sample is from pre-treatment or post-treatment with G100.

### Statistical Analysis

**Figure 1:**
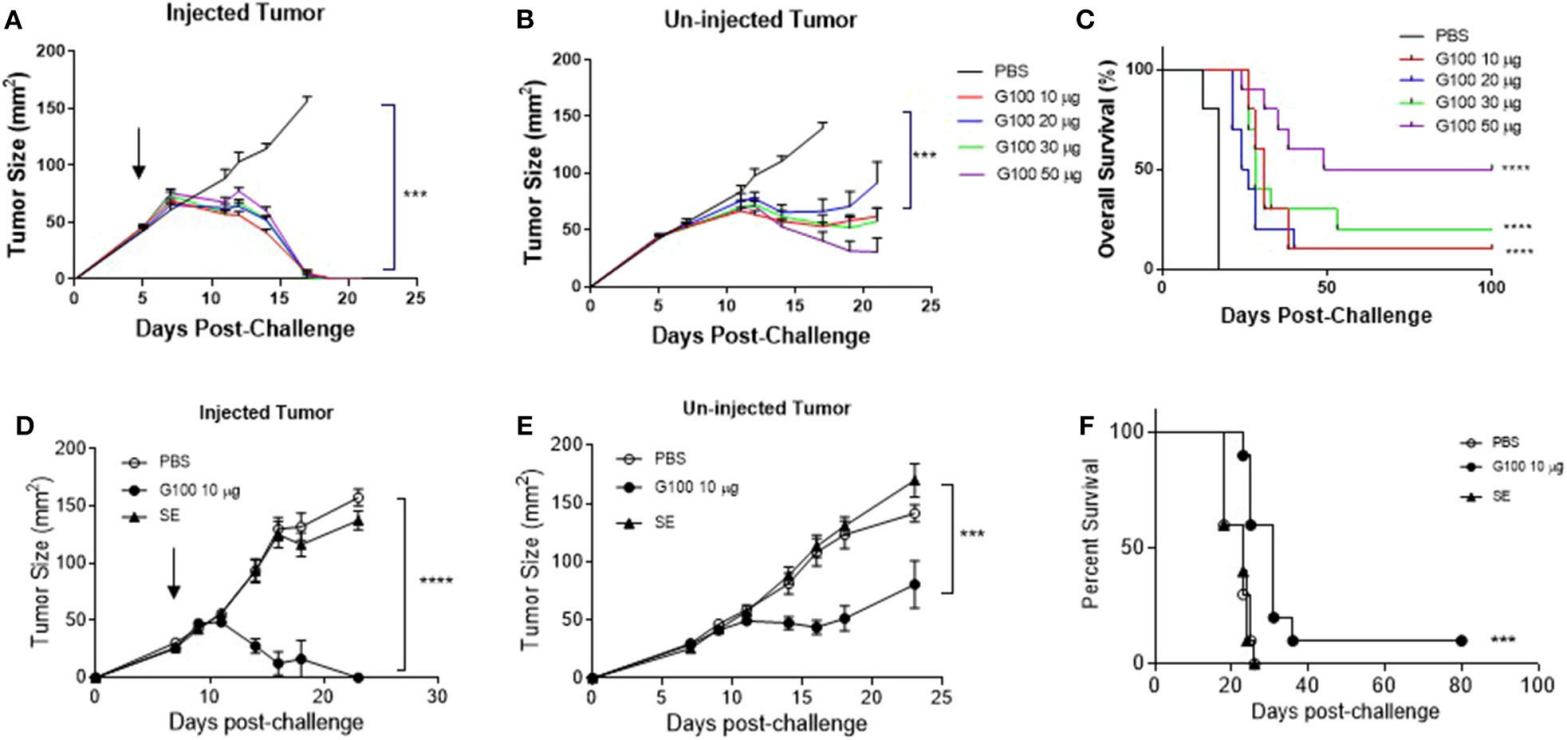
G100 cures established murine A20 lymphomas and the anti-tumor effect depends on tumor cell TLR4 expression. **(A,B)** Tumor growth curves of G100-injected or contralateral un-injected A20 tumors. Five million A20 were implanted subcutaneously (s.c.) into Balb/c mice (*n* = 5/group) on both flanks. Intratumoral (i.t.) injection of G100 (10, 20, 30, or 50 μg GLA-SE) or PBS started on day 5 [indicated by the arrow on **(A)**] when the tumors were palpable (average size ~40 mm^2^), three times per week, for a total of 9 injections. Mice were sacrificed when any tumor size reached over 150 mm^2^. **(C)** Survival curves in mice with bilateral A20 tumors treated with different doses of GLA-SE and PBS control. All the G100-treated mice (10, 20, 30, or 50 μg GLA groups) had significant longer survival than control (PBS treated) mice (*p* < 0.0001, adjusted *p*-values using Bonferroni method). The survival in the high dose (50 μg GLA) G100 group was significantly better than that in low dose group (10 μg) (*p* = 0.03). **(D,E)** Tumor growth curves in mice treated with G100 and two controls, PBS and SE, for both **(D)** injected tumors, and **(E)** un-injected tumors. ***p < 0.001, ****p < 0.0001 based on tumor size on day 18. There was no difference in growth kinetics between PBS and SE groups for both the injected and un-injected tumors. The arrow in **(D)** indicate treatment start time. **(F)** Survival curves in mice treated with both PBS and SE controls. ***p < 0.001 between G100 and SE or PBS. Again, there was no difference between SE and PBS.

Statistical analysis was performed using SAS version 9.4 & GraphPad Prism (GraphPad software, San Diego, CA). Two-tailed Student *t*-test was used to analyze differences between treatment groups in tumor size (last measurement shown in figure) and tumor infiltrating lymphocytes (FACS). ANOVA was used to analyze the treatment time- and dose- dependent response of GLA on cell growth inhibition and induction of apoptosis and necrosis in A20 cells. Kaplan-Meier plots were used to analyze the mouse survival data in A20 lymphoma model. Comparison between survival curves was done using the log-rank test. Conclusions from sensitivity analysis using Fleming-Harrington test with parameters (1, 0) to address late effect are consistent with the main analysis using Log rank test (in Figure 1C). Bonferroni adjustment was used to control type 1 error for multiple comparisons. A *p* < 0.05 was considered statistically significant.

## Results

### Anti-tumor Effects of G100 in the A20 Model Depend on Tumor Cell TLR4 Expression

**Figure 2:**
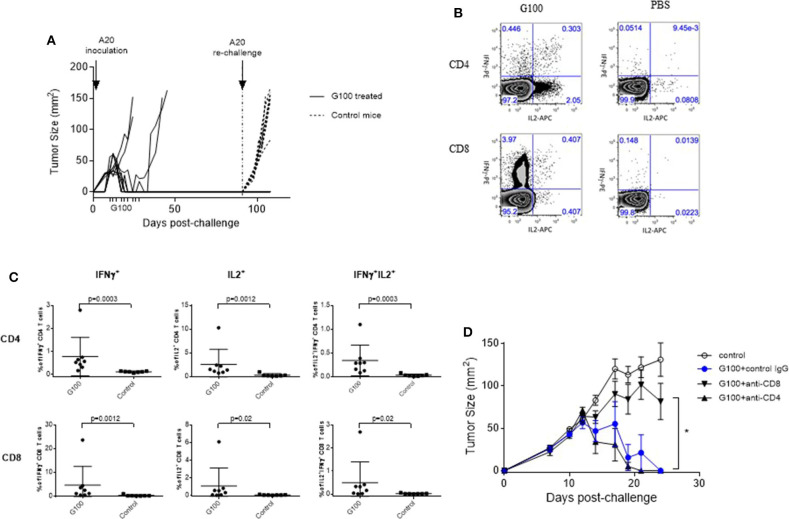
G100 induces CD8 T cell-mediated tumor protection and memory responses. **(A)** Rejection of A20 tumor re-challenge in G100-treated mice. On Day 7 after A20 tumor cell inoculation, mice with palpable A20 tumors started receiving GLA-SE treatment (10 μg, 3x/week, for 3 weeks), as indicted on the x-axis. Six out of 10 treated mice had complete tumor regression and received a second tumor challenge on the opposite side of the original tumor at 3 months after the initial tumor inoculation (indicated by vertical dotted line on day 90). All these mice rejected the secondary tumor challenge, demonstrating the development of long-term immunity. Untreated mice developed tumor rapidly after A20 challenge, as shown in tumor growth curves with dotted lines. **(B)** Splenocytes were collected from G100-treated mice and naïve Balb/c mice (*n* = 8 per group) and stimulated with irradiated A20 tumor cells at a 10:1 ratio with the addition of brefeldin A and cultured overnight. Then the cells were then either surface stained or permeabilized and stained with antibodies against IFNγ, IL2, and TNFα and analyzed by FACS. Shown are representative dot plots showing IL-2 and IFNγ production in CD4 and CD8 T cells in splenocytes co-cultured with A20 tumor cells. For data analysis, cells were first gated as singlets based on FSC and SSC. Then the singlet cells were gated based on a viability dye. The live cells were gated for CD3+ T cells, which were further separated into CD4 and CD8 T cells. **(C)** Summary graphs showing the levels of cytokine positive cells in CD4 and CD8 T cells from GLA-treated or control naïve mice. **(D)** Tumor growth curves (mean ± sem) in mice that were treated with PBS, G100 (10 μg GLA, 2%SE /mouse, 3 times/week for 3 weeks), or G100 plus CD4 or CD8 T cell depletion. The depletion efficiency was more than 95% as measured by FACS (data not shown). Depletion of CD8 T cells but not CD4 T cells significantly abrogated the anti-tumor effects of G100. *p < 0.05 between G100 and G100 + CD8 depletion group.

**Figure 3:**
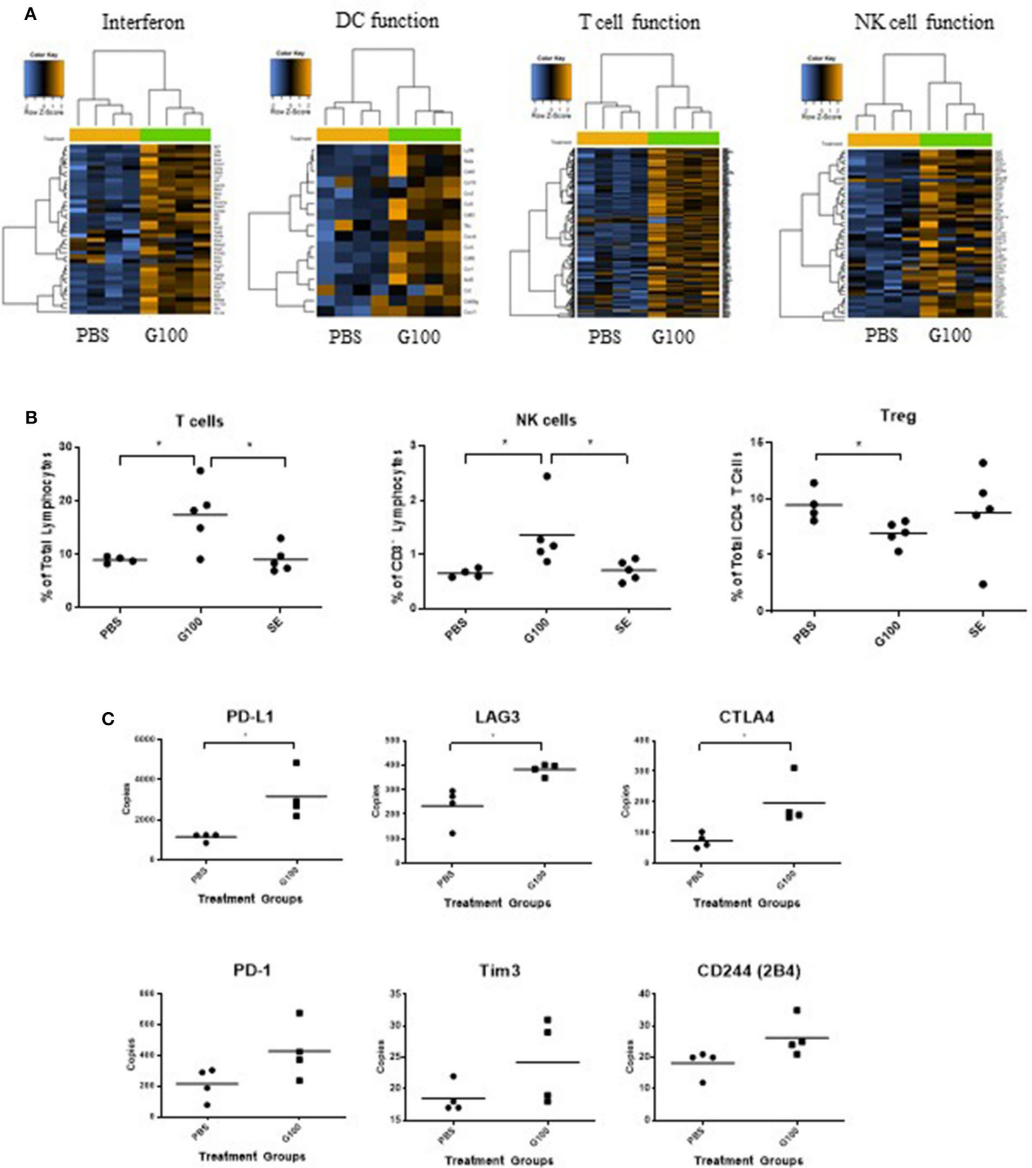
G100 inflames TME of A20 tumors. **(A)** A20 tumors (*n* = 4 mice/group) treated with G100 (10 μg/ml GLA, 2%SE) for 1 week (3 injections, every other day) were collected for gene expression analysis by Nanostring. Multiple immune response pathways, including type 1 interferon signaling, DC function, T cell, and NK cell functions, were all activated. **(B)** Tumor infiltrating lymphocytes (TIL) were collected for Flow cytometry analysis and stained with antibodies for CD3, CD4, CD8, Foxp3, and NKp46. G100 treatment induced significant increase in T cells and NK cells and a decrease in T_reg_ among CD4 T cells. SE treatment was no different from PBS control in any of the markers. **(C)** Gene expression levels of T cell exhaustion markers in G100-treated and non-treated tumors (*n* = 4 mice/group). *p < 0.05 by student *t*-test.

As shown in Figures 1A,B, G100 treatment (10–50 μg, intratumoral 3x/week, 7–9 doses total) inhibited the growth of both injected (treated) and un-injected tumors (abscopal lesions) in the A20 murine lymphoma model. The overall survival (OS) of bilaterally challenged mice was significantly prolonged in all treatment groups receiving G100 (10, 20, 30, or 50 μg GLA/2%SE), as compared to PBS treated mice (*p* < 0.0001, Figure 1C), with a dose response observed and greatest survival (~50%) in the 50 μg group. To investigate whether the stable emulsion (SE) formulation we used for G100 had any effects on its own, mice were treated with G100 (10 μg in 2%SE), SE (2%), or control PBS. As shown in Figures 1D–F, injection of SE alone had no tumor inhibitory effect and showed no difference from PBS. All surviving mice rejected tumor re-challenge 3 months after primary challenge, demonstrating a robust memory response (Figure 2A). Mice treated with G100 also had significantly higher levels of tumor antigen-specific CD4 and CD8 T cells that secrete IFNγ and IL2 after *in vitro* stimulation with A20 cells (Figures 2B,C). Selective depletion studies further demonstrated that the anti-tumor effect was dependent on CD8 T cells (Figure 2D). G100 activated multiple genes related to DC function, T cell and NK cell function in the A20 TME (Figure 3A and Supplementary Table 1) and FACS analysis of tumor infiltrating lymphocytes (TIL) showed significantly (*p* < 0.05) increased (~2-fold) percentages of T cells and NK cells, with decreased (~30%) Tregs in G100-treated mice (Figure 3B). Most exhaustion markers tested (PD-1, PDL1, LAG3, TIM3, CD244, CTLA4) were upregulated in A20 tumors after G100 treatment (Figure 3C).

**Figure 4:**
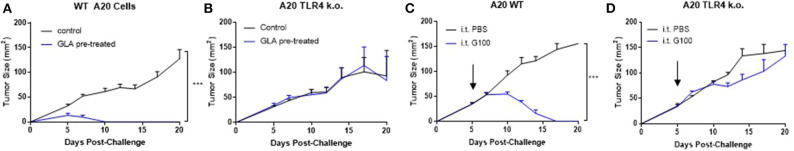
GLA has anti-tumor effects in A20 WT but not A20 TLR4 ko tumor cells *in vitro* and *in vivo*. **(A,B)** Pre-treatment with GLA-AF (incubating cells in medium containing GLA-AF (5 μg /ml) *in vitro* for 48 h prior to subcutaneous implantation in mice inhibited the growth of wild type (WT), but not TLR4 knockout (k.o.) A20 tumor growth after inoculation. **(C,D)** IT G100 treatment inhibited the growth of WT but not TLR4 k.o. A20 tumors. IT injection of G100 (10 μg GLA-SE, 3x/week) started on Day 7 after the tumors were established and lasted for 3 weeks. Shown are average tumor sizes (mean ± sem) for each treatment groups (*N* = 5/group). ***p < 0.001 by student *t-*test.

Since A20 is derived from murine B cells which are known to express TLR4, we investigated the importance of TLR4 expression on tumor cells by generating a biallelic TLR4 knockout A20 cell line. To separate direct GLA effects on tumor cells from effects of GLA on immune cells in the TME, we pretreated A20 WT cells or A20 TLR4 k.o. cells with GLA-AF (5 μg /ml) *in vitro* for 48 h prior to subcutaneous implantation. As shown in Figures 4A,B, GLA-pretreated A20 WT cells did not establish tumors, whereas the TLR4 k.o. cell line was not affected by GLA pretreatment and established tumors with the same growth kinetics as A20 WT cells. A side-by-side comparison of IT G100 effect on WT A20 tumor vs. TLR4 k.o. tumors showed that the *in vivo* anti-tumor effects of G100 were entirely dependent on expression of TLR4 (Figures 4C,D).

### GLA Induces APC Functions and Apoptosis in A20 Cells

**Figure 5:**
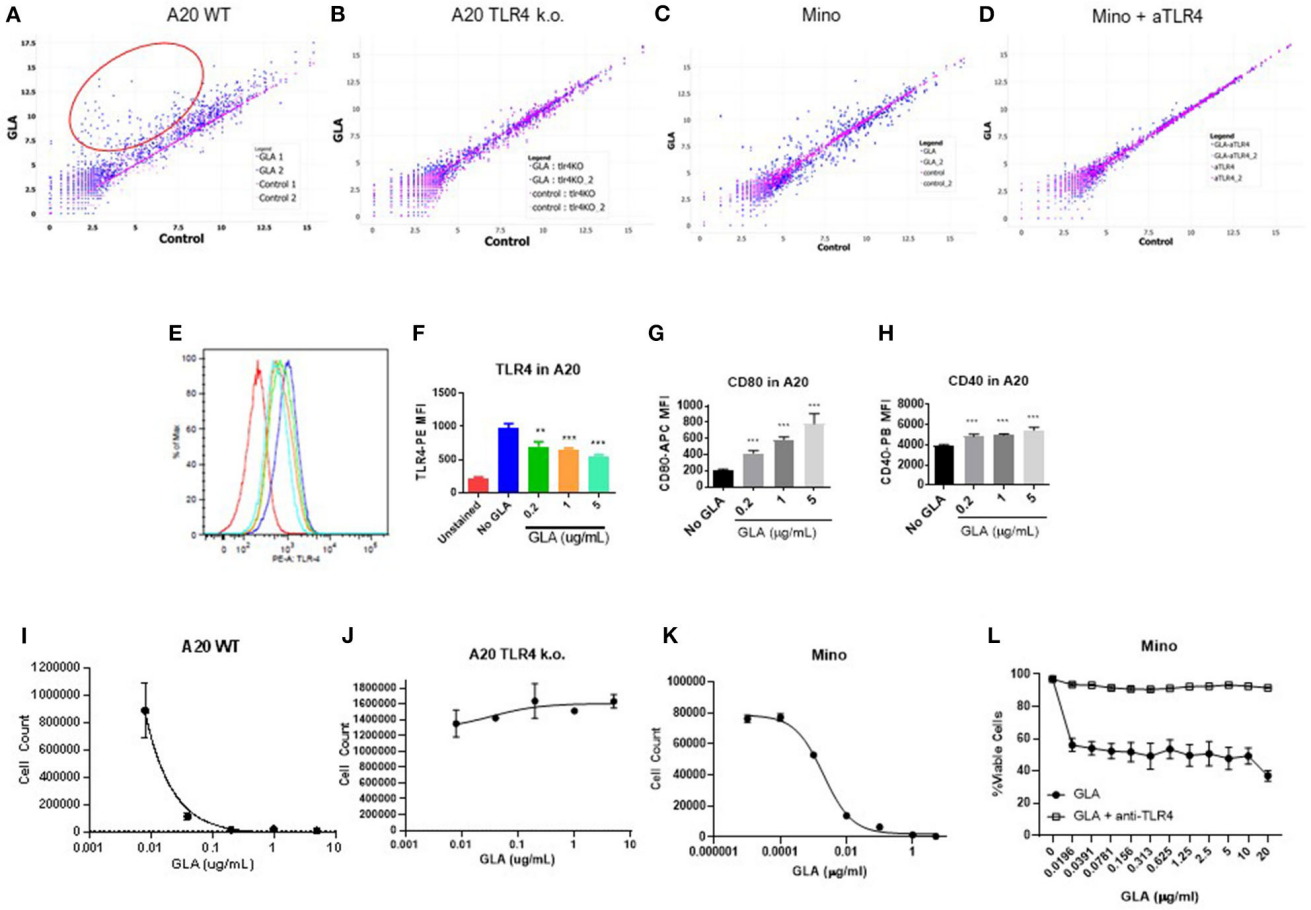
GLA has direct effects on murine and human lymphoma cells by inducing activation and cell death. **(A–D)** Scatter plot of gene expression analysis in murine or human lymphoma cells. Each dot represents the expression level of a particular gene in the control group (x-axis) or GLA-treated (y-axis) group. The full list of genes with differential expression is available in Supplementary Tables 2–5. **(A,B)** A20 WT or TLR4 k.o. cells were treated with GLA-AF (5 μg/mL, 48 h) before gene expression analysis by Nanostring using the mouse panCancer immune profiling panel. **(A)** Scatter plot of gene expression levels in GLA- or control PBS- treated WT A20 cells. **(B)** Scatter plot of gene expression levels in GLA- or PBS- treated TLR4 k.o. A20 cells. The red circle marks genes that are significantly induced by GLA treatment. Two independent samples are included in each treatment group. **(C)** Mino lymphoma cells were treated with GLA-AF (5 μg/mL, 48 h) before gene expression analysis by Nanostring using the human panCancer immune profiling panel. **(D)** TLR4 was blocked by adding anti-TLR4 mAb (10 μg/mL) 15 min before GLA treatment and gene expression analysis. **(E–H)** Direct effects of GLA in A20 tumor cells *in vitro*. A20 tumor cells were treated with GLA (0.2, 1, or 5 μg/ml GLA-AF, 48 h) before staining with anti-TLR4/MD2, anti-CD80, or anti-CD40. Shown are overlay histogram of TLR4 staining in A20 cells **(E)**, and summary graphs of mean fluorescent intensity (MFI) of TLR4, CD80, and CD40 expression in GLA-treated or control A20 cells **(F–H)**. Treatment was done in duplicate. Each column indicates mean ± sem of duplicate wells. **(I,J)** Dose-dependent inhibition of the growth of WT but not TLR4 k.o. A20 cells by GLA. The cells were treated with serial dilution of GLA (0.01–5 μg/mL) for 96 h before the cells were stained with Live/Dead-NIR and evaluated by FACS. Shown are mean ± sem of cell numbers in two duplicate treatment wells. **(K)** Dose-dependent inhibition of the growth of Mino lymphoma cells. The cells were treated with serial dilution of GLA for 96 h before the cells were stained with Live/Dead-NIR and evaluated by FACS. **(L)** The addition of anti-TLR4 mAb blocked the growth inhibitory effects of GLA in Mino cells. **p < 0.01, ***p < 0.001.

**Figure 6:**
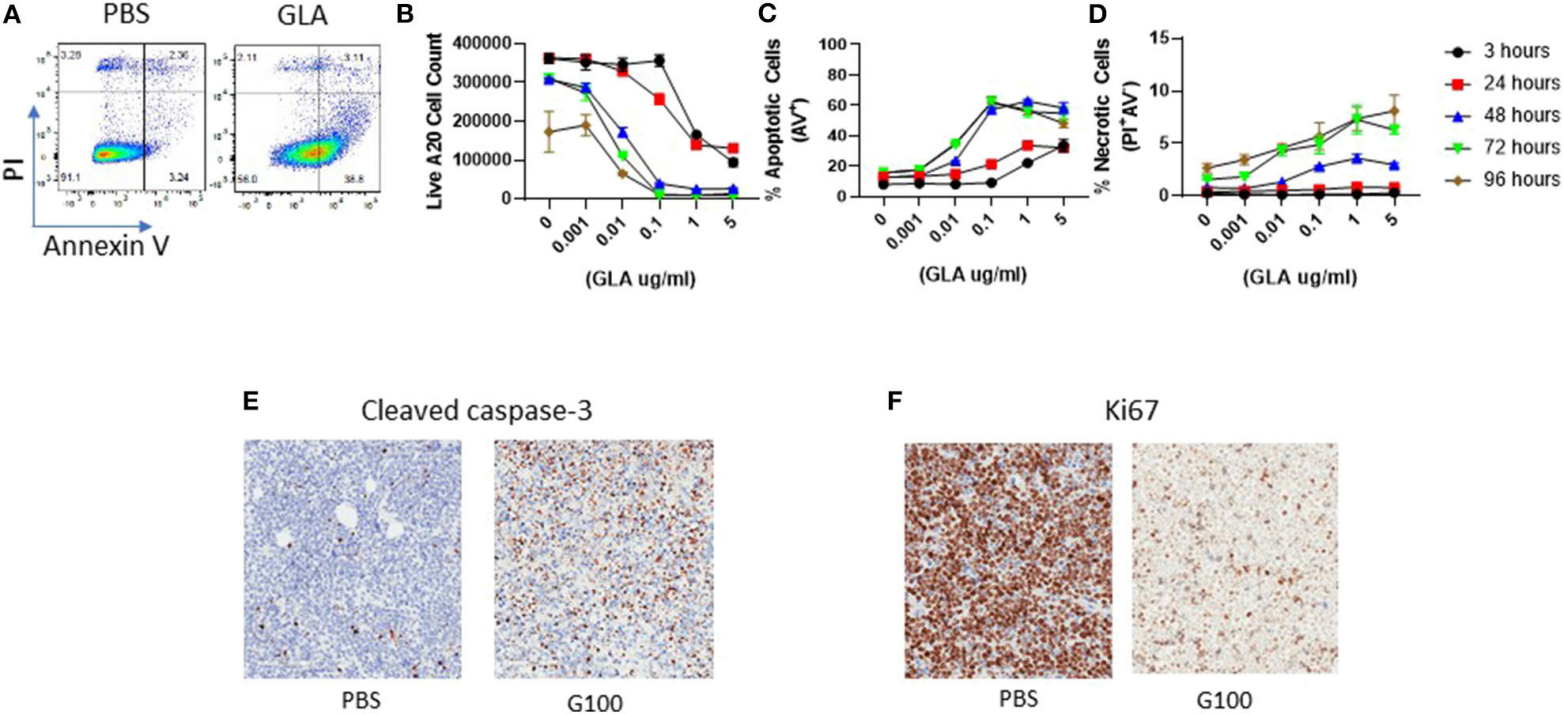
Induction of A20 tumor cell apoptosis by GLA *in vitro* and *in vivo*. **(A)** Representative FACS graphs showing Annexin-V and PI staining in control or GLA-treated A20 cells. A20 cells were treated with GLA (5 μg/mL) for 48 h before staining with Annexin-V and PI. **(B–D)** Dose and time- dependent effect of GLA on the number of live A20 cells **(B)** and induction of apoptosis **(C)** and necrosis **(D)**. A20 cells were treated with serial dilutions of GLA-AF for 3, 24, 48, 72, or 96 h before the cells were stained with Annexin-V/PI and analyzed by flow cytometry. Each data point represents the mean ± sem of cell number or percentage of apoptotic or necrotic cells in two replicate wells. **(E,F)** *in vivo* G100 treatment induced cleaved caspase-3, an apoptosis marker, and decreased the expression of Ki67, a proliferation marker, in A20 tumor cells. A20 tumors were collected after three intratumoral injections of G100 (10 μg) or control PBS for IHC analysis. Brown dot indicates tumor cells that are positive for cleaved caspase-3 **(E)** or Ki67 **(F)**.

To address the potential direct effect of GLA on tumor cells, we examined both gene expression and phenotypic changes in WT and TLR4 k.o. A20 cells after GLA treatment. As shown in Figures 5A,B, treatment with GLA-AF (5 μg/mL, 48 h) resulted in induction of multiple immune-related genes in WT, but not TLR4 k.o. A20 cells. The upregulated genes include co-stimulatory molecules (CD40, CD80, CD86) and MHCII (H2-DMb2, H2-Eb1, H2-Dma, Supplementary Tables 2, 3). Flow cytometry analysis showed a dose-dependent reduction of TLR4 expression (Figures 5E,F) and dose-dependent induction of CD40 and CD80 by GLA-AF in WT A20 cells (Figures 5G,H). The induction of genes related to B cell activation and APC function was also observed in human Mino cells, a TLR4 expressing mantle cell lymphoma cell line (Figure 5C and Supplementary Table 4). Gene induction was blocked when cells were pretreated with an anti-human TLR4 mAb (Figure 5D and Supplementary Table 5). Prolonged exposure to GLA-AF (0.01–5 μg/mL, 96 h) resulted in dose-dependent decrease in cell number in WT but not TLR4 k.o. A20 cells (Figures 5I,J). The decrease in cell number and viability by GLA treatment was also seen in Mino cells and the effect was blocked by anti-TLR4 mAb (Figures 5K,L). The decrease in cell number in A20 cells after GLA treatment is consistent with induction of apoptosis (Figure 6A). Time course studies further showed that the induction of apoptosis and to a lesser degree also necrosis by GLA-AF in A20 cells was both dose- and time- dependent (Figures 6B–D). The IC_50_ at 48 h was ~ 0.01 μg/mL. The ability of GLA to induce apoptosis was also observed *in vivo*. As shown in Figures 6E,F, after three injections of G100 or control PBS, G100-treated A20 tumor had significantly increased expression of cleaved caspase-3, an apoptosis marker, and decreased expression of Ki67, a proliferation marker.

**Figure 7:**
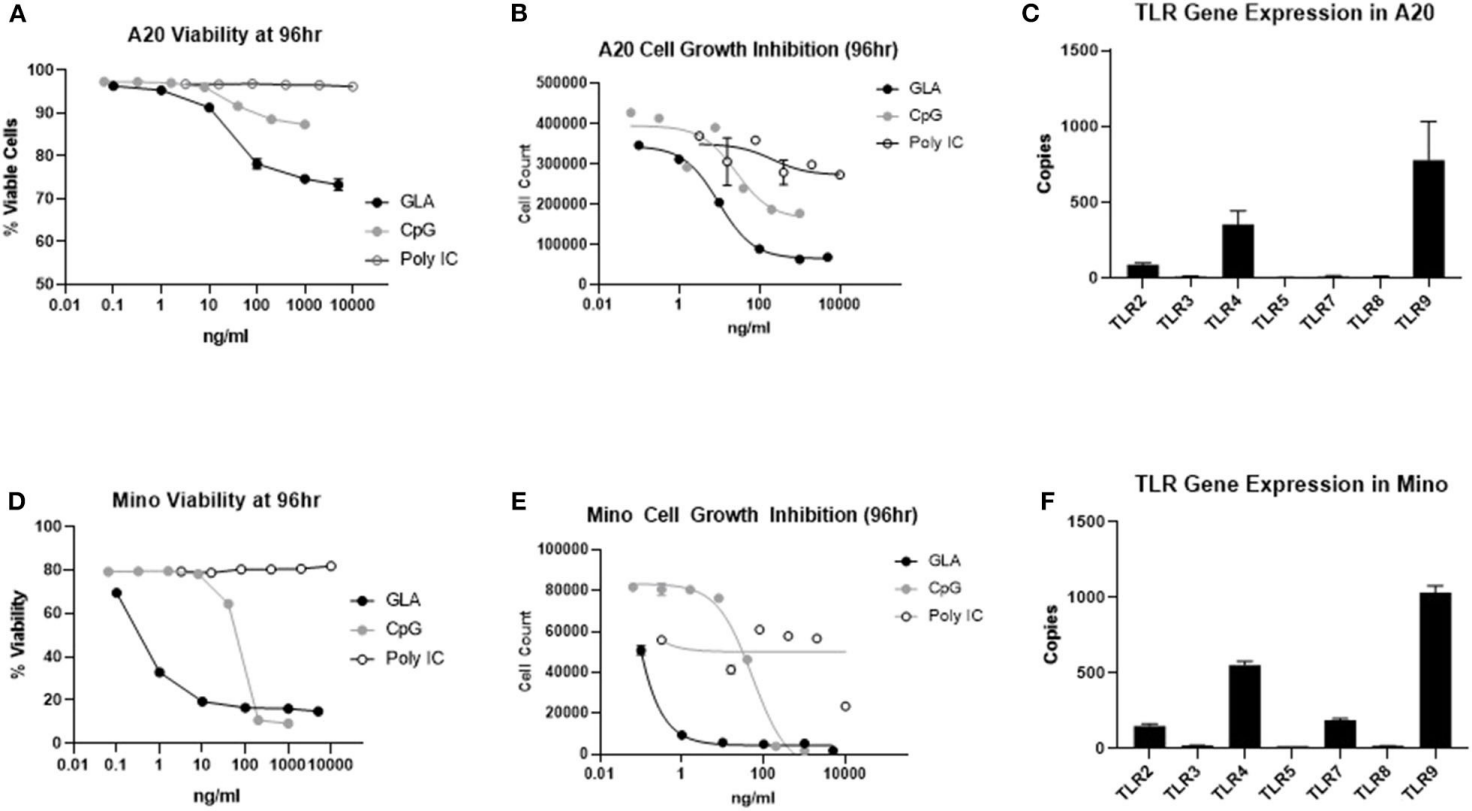
A20 murine lymphoma and Mino human lymphoma cell lines are more sensitive to TLR4 agonist than TLR3 and TLR9 agonists. **(A)** GLA significantly inhibited the viability of A20 cells *in vitro*. Shown is the average viability of A20 cells (from two duplicate wells) at the given concentrations of GLA, CpG, or poly I:C. **(B)** GLA is more potent than poly I:C and CpG in inhibiting the growth of A20 cells. Shown are the average number of viable cells (y-axis) from two duplicate treatment wells at the given concentrations of GLA, CpG, or poly I:C (x-axis). **(C)** Expression level of different TLRs in A20 cells as determined by Nanostring gene expression assay using the panCancer Immune Profiling panel. Each bar indicates the expression level (mean ± sem) of a specific TLR gene in two independent RNA samples from A20 cells. **(D)** GLA significantly inhibits the viability of Mino cells. Shown is the average viability of Mino cells (from two duplicate wells) at the given concentrations of GLA, CpG, or poly I:C. **(E)** GLA is more potent than poly I:C and CpG in inhibiting the growth of Mino cells. Shown are the average number of viable cells (y-axis) from two duplicate treatment wells at the given concentrations of GLA, CpG, or poly I:C (x-axis). **(F)** Expression level of different TLRs in Mino cells as determined by Nanostring gene expression assay using the panCancer Immune Profiling panel. Each bar indicates the expression level (mean ± sem) of a specific TLR gene in two independent RNA samples from Mino cells.

To evaluate whether the direct effect on A20 tumor cells could also be induced by other TLR agonists currently used for intratumoral therapy, we treated A20 cells side-by-side with the TLR3 agonist poly I:C, TLR9 agonist ODN2006, or GLA-AF. As shown in Figure 7A, direct cytotoxicity on A20 cells was observed after treatment with GLA and CpG ODN2006, but not after treatment with poly I:C. Dose-response curves showed that A20 cells were most sensitive to GLA as compared to other TLR agonists (Figure 7B). Gene expression analysis showed that untreated A20 cells mainly expressed TLR4 and TLR9, but not other TLRs (Figure 7C). Overall, results obtained with human Mino cells were very similar (Figures 7D–F).

## Discussion

B-cells can play an important role function as antigen-presenting cells (APC), for example in the setting of prophylactic vaccination, and it is currently being investigated whether B-tumor cells possess APC function that can be exploited therapeutically to induce clinically relevant anti-tumor responses (19, 20). To this end, stimulation with anti-CD40 antibody, CD40 ligand, or CpG has been shown to enhance the antigen presenting capacity of murine lymphoma cell lines and human tumor B cells via enhancing the expression of MHC-II, costimulatory molecules (mostly CD86 but also CD80) and adhesion molecules (CD54) (21–24).

Toll-like receptor 4 (TLR4) is widely expressed on immune cells in the mouse, especially macrophages and dendritic cells, as well as on B-cells and B-cell lymphomas. Dendritic cells (DC) are activated and matured by TLR4 agonists, and TLR4 engagement has been shown to promote cross-presentation in CD103+ murine DC (25). Stimulation of TLR4 also plays an important role in B-cell development, proliferation and induction of effector functions, such as secretion of antibodies and cytokines, immunoglobulin class-switch recombination, upregulation of co-stimulatory molecules (CD40, CD80), and activation of antigen presentation in the lymph node (26). Because we have previously shown safety, immunological activity and clinical benefit of IT G100 treatment in two small phase 1 trials in soft tissue sarcoma and Merkel cell carcinoma patients, we sought to generate preclinical data in the A20 model that support clinical studies in follicular lymphoma patients (17, 27).

We demonstrate here in a bilateral model of A20 lymphoma that IT treatment of only one tumor with the synthetic TLR4 agonist GLA at the highest dose evaluated (50 μg) cured ~ 50% of mice, indicating the induction of a systemic immune response. This was confirmed by showing that the anti-tumor effects were mediated by tumor-specific splenic CD8 T-cells and that mice surviving a primary A20 challenge were completely protected against re-challenge 3 months later. A20 cells have been shown to be phenotypically similar to mature human B-cell lymphomas, expressing high levels of MHC class I and class II molecules and moderate levels of the costimulatory molecule B7-2 (CD86), and to present both exogenous and endogenous antigen (28). However, despite their intrinsic antigen-presenting capabilities, A20 tumors fail to be eliminated spontaneously and tumor-specific T cells are tolerized rather than activated *in vivo* (29). In contrast, G100- treated A20 tumors showed upregulation of genes related to DC activation, antigen presentation, NK cell activation, T cell activation and other innate and adaptive immune functions, in line with previous reports characterizing in detail the innate immune activation with GLA in muscle and draining lymph nodes of mice (12, 30). Indicative of IFNγ-mediated adaptive immune resistance in the TME, upregulation of multiple checkpoint molecules, such as PD1, LAG3, TIM3, CTLA4, and PD-L1 in the TME was also observed in G100 treated tumors.

To investigate the possibility that G100-stimulated B-tumor cells would participate in the induction of antitumor responses, we first determined that the cells had an intermediate level of TLR4 expression at baseline as measured by flow cytometry. Next, we showed that *in vivo* GLA treatment of A20 cells upregulated the costimulatory markers CD80, CD86, and CD40, as well as MHCII, indicating the induction of APC functions. Interestingly, prolonged GLA incubation (48–96 h) also induced genes involved in apoptosis and cell death (caspase-1, caspase-3, Fas) and a significant reduction in cell numbers. Pre-treatment of A20 cells with GLA resulted in failed tumor establishment when implanted into mice. Taken together, these results suggested that GLA-mediated signaling induced APC functions as well as direct cytotoxicity, which likely both contributed to the observed antitumor effects. To test this hypothesis, we generated an A20 cell line with biallelic knockout of the TLR4 gene. Strikingly, both the direct effect of GLA on tumor cells (upregulation of activation markers, induction of apoptosis) and the clinical effect of intratumoral G100 injection (tumor shrinkage) were completely abrogated in the TLR4 k.o. model. This is unequivocal evidence that immune functions of A20 lymphomas cells can be harnessed directly to induce immune responses that eliminate tumors, without the need for additional therapeutic modalities to induce tumor cell death or cross-presentation. Previously, treatment of A20 tumors with intratumorally injected synthetic oligodeoxynucleotides (ODNs) containing unmethylated CpG motifs (CpG), which are strong activators of toll-like receptor 9, was shown to have similar clinical efficacy as G100, however, knockout of TLR9 in tumor cells or the host animal reduced sensitivity to CpG but did not eliminate it completely (31). We quantified the expression of toll-like receptors 2, 3, 4, 5, 7, 8, and 9 in A20 tumors by RNA profiling, showing that TLR9 was expressed highest, followed by TLR4 and TLR2, whereas no TLR3 expression was detectable. This would explain why a recent publication reported that using a three-component *in situ* vaccine (ISV), consisting of the DC-activator Flt3 ligand, low-dose gamma irradiation and the toll-like receptor 3 (TLR3) agonist poly-ICLC, A20 tumors could only be cured if cross-presenting CD103+ dendritic cells were activated intratumorally (32). In summary, our experiments show that the effects of IT treatment of A20 tumors with a TLR4 agonist depends critically on the direct activation of the tumor cells themselves, in particular of antigen presenting pathways, which presumably turns them into target cells with increased susceptibility for T cell mediated killing.

There are limitations to our current study. It is recognized that although we have shown that both the direct effect of TLR4 agonist on tumor cells and CD8 T cell-mediated immune effects are necessary for the anti-tumor effects of GLA, a clear distinction between the two needs further investigation. To this end, when CD8 T cells isolated from tumor rejection mice are simultaneously transferred with A20 tumor cells to naïve recipient mice, no tumors formed (data not shown). Additional studies analyzing contralateral rejection of TLR4 KO A20 tumors upon treatment of WT A20 tumors, or implantation of WT A20 tumors in TLR4 k.o. mice would also help further delineate the role of immune-mediated effects. Additional studies on the pharmacokinetics (PK) of GLA after *in vivo* administration will also be informative to guide further optimization on formulation (oil concentration) and dosing regimen for anti-tumor effects. Preliminary studies have shown that after a single dose of intratumoral GLA-SE injection (10 μg), GLA was detectable in tumors at 96 h post-injection, but how the concentration in tumor compares to concentration used in our *in vitro* studies need further evaluation. Whether increasing the percentage of SE, which has known Th2-type immunomodulatory effects, will impact anti-tumor effect also remains to be investigated.

TLR4 staining by immunohistochemistry (IHC) has been examined in a number of hematologic malignancies and increased expression can be demonstrated on tumor cells in diffuse large B-cell lymphoma (DLBCL), multiple myeloma, and B acute lymphoblastic leukemia (B-ALL) (33, 34). We show here that the TLR4 positive human B-lymphoma cell line Mino responds to TLR4 stimulation in a very similar fashion to the murine A20 tumor cell line. TLR4 expression on tumor cells may therefore be biomarker for identifying patients with B-cell lymphomas susceptible to IT G100 treatment. To this end, we are currently performing a phase 1/2 trial of IT G100 in patients with follicular low-grade non-Hodgkin's lymphoma, with or without concurrent systemic anti-PD1 (Pembrolizumab) treatment (NCT02501473).

## Data Availability Statement

The datasets presented in this study can be found in online repositories. The names of the repository/repositories and accession number(s) can be found below: the NCBI Gene Expression Omnibus (GSE150457).

## Ethics Statement

The studies involving animals were reviewed and approved by the IACUC committee of the Infectious Disease Research Institute, Seattle.

## Author Contributions

HL and JM designed the study and wrote the paper. HL and AB performed the experiments. MC performed the statistical analysis. All authors contributed to the article and approved the submitted version.

## Conflict of Interest

HL, JM, AB, and MC were full time employees and shareholders of Immune Design at the time of performing the study. The authors declare that this study was fully funded by Immune Design. The funder fully designed and executed the study.
